# AST-120 improved uremic pruritus by lowering indoxyl sulfate and inflammatory cytokines in hemodialysis patients

**DOI:** 10.18632/aging.205580

**Published:** 2024-02-21

**Authors:** Chia-Chao Wu, Ya-Chung Tian, Chien-Lin Lu, Ming-Ju Wu, Paik-Seong Lim, Yi-Wen Chiu, Ko-Lin Kuo, Shou-Hsuan Liu, Yu-Ching Chou, Chien-An Sun, Yi-Chou Hou, Kuo-Cheng Lu

**Affiliations:** 1 Division of Nephrology, Department of Internal Medicine, Tri-Service General Hospital, National Defense Medical Center, Taipei 11490, Taiwan; 2 Department and Graduate Institute of Microbiology and Immunology, National Defense Medical Center, Taipei 11490, Taiwan; 3 Kidney Research Center, Department of Nephrology, Chang Gung Memorial Hospital, Taoyuan City 33303, Taiwan; 4 School of Medicine, College of Medicine, Fu Jen Catholic University, New Taipei City 24205, Taiwan; 5 Division of Nephrology, Department of Medicine, Fu Jen Catholic University Hospital, New Taipei City 24352, Taiwan; 6 Division of Nephrology, Department of Internal Medicine, Taichung Veterans General Hospital, Taichung 40705, Taiwan; 7 Department of Post-Baccalaureate Medicine, College of Medicine, National Chung Hsing University, Taichung 40227, Taiwan; 8 Division of Nephrology, Department of Internal Medicine, Tungs’ Taichung Metroharbour Hospital, Taichung 43503, Taiwan; 9 Division of Nephrology, Department of Internal Medicine, Kaohsiung Medical University Hospital, Kaohsiung 80756, Taiwan; 10 Faculty of Medicine, Kaohsiung Medical University, Kaohsiung 80708, Taiwan; 11 Division of Nephrology, Department of Medicine, Taipei Tzu Chi Hospital, Buddhist Tzu Chi Medical Foundation, New Taipei City 23142, Taiwan; 12 School of Medicine, Buddhist Tzu Chi University, Hualien 97004, Taiwan; 13 School of Post-Baccalaureate Chinese Medicine, Buddhist Tzu Chi University, Hualien 97004, Taiwan; 14 Department of Nephrology, Department of Internal Medicine, Chang Gung Memorial Hospital, Taoyuan City 33303, Taiwan; 15 School of Public Health, National Defense Medical Center, Taipei 11490, Taiwan; 16 Department of Public Health, College of Medicine, Fu-Jen Catholic University, New Taipei City 24205, Taiwan; 17 Big Data Research Center, College of Medicine, Fu-Jen Catholic University, New Taipei City 24205, Taiwan; 18 Division of Nephrology, Department of Internal Medicine, Cardinal-Tien Hospital, New Taipei City 23155, Taiwan; 19 Division of Nephrology, Department of Medicine, Fu-Jen Catholic University Hospital, School of Medicine, Fu-Jen Catholic University, New Taipei City 24352, Taiwan

**Keywords:** protein bounded uremic toxin, uremic pruritus, AST-120, indoxyl sulfate, end-stage renal disease

## Abstract

Background and hypothesis: Pruritus is a common and distressing symptom that affects patients with chronic kidney disease. The concentration of protein bounded uremic toxin was associated with the uremic pruritus. The aim is to assess the efficacy of AST-120 for uremic pruritus in hemodialysis patients.

Materials and Methods: The participants were enrolled and then divided into the AST-120 treatment group and control group with a ratio of 2:1. All participants underwent pre-observation screenings two weeks before the study with three visits. In the treatment phase (week 1 to week 4), the treatment group added 6g/day of AST-120 along with routine anti-pruritic treatment. Visual analog scale (VAS) and biochemical parameters were measured.

Results: The VAS score began to be lower in the AST-120 treatment group after the 5th visiting (*p* < 0.05). The reduction in indoxyl sulfate (IS) at 5th week along with TNF-alpha. The reduction ratio of indoxyl sulfate correlated with reduction of parathyroid hormone.

Conclusion: This study has demonstrated that the four-week treatment of AST-120 decreased the severity of uremic pruritus in patients with ESRD. The concentration of IS and TNF-alpha decreased in the AST-120 treatment group. The reduction of iPTH correlated with the reduction of IS in the AST-120 treatment.

## INTRODUCTION

Pruritus is a common and distressing symptom that affects patients with CKD [1]. The pathogenesis of uremic pruritus remains unclear, but it is believed that inflammation may have a role in its development. These increased inflammatory markers may partly explain the association between low hemoglobin levels and a higher prevalence of pruritus, as low hemoglobin levels are often associated with inflammatory states [2]. The pathophysiology of uremic pruritus involves various factors. These include subclinical or overt uremic neuropathy, skin or nerve inflammation associated with chronic systemic inflammation in kidney failure, and an increase in the activity of μ-opioid receptors due to kidney failure [3].

A recent international study found that approximately 40% of patients with end-stage renal disease (ESRD) undergoing hemodialysis experience moderate-to-extreme pruritus. This condition is associated with a higher prevalence of comorbid conditions, worse biochemical profiles, poorer mental and physical quality of life, higher probability of depression, and poorer sleep quality and survival [1]. More recently, this prevalence was shown to range from 26% in Germany to 48% in the United Kingdom [4]. Several studies have provided evidence linking pruritus with adverse effects on kidney disease burden, health-related quality of life, and sleep disturbances in patients undergoing dialysis. These findings suggest that pruritus is associated with negative outcomes in individuals with kidney disease [5]. To date, the management of uremic pruritus has been classified into medical and non-medical interventions, but there is still limited understanding of the effectiveness of various treatments in CKD/ESRD patients [3].

Protein bounded uremic toxins, such as indoxyl sulfate (IS) and p-cresol or p-cresyl sulfate (PCS), accumulate significantly in the organs of chronic kidney disease (CKD) patients. These toxins have the potential to induce inflammatory reactions, enhance oxidative stress, and worsen renal function by causing glomerular sclerosis and interstitial fibrosis. The concentration of protein bounded uremic toxin is associated with the development of pruritus [6]. IS and PCS are uremic toxins with similar protein binding, dialytic clearance, and proinflammatory features [7]. Oral sorbent AST-120 (AST-120; KREMEZIN^®^, Kureha, Tokyo, Japan) can mitigate these effects by reducing the levels of uremic toxins in CKD patients. AST-120 achieves this by adsorbing the precursors of IS and PCS generated through amino acid metabolism in the intestine [8]. This impedes oxidative stress, slows the progression of cardiovascular and renal diseases, and improves bone metabolism in patients with CKD [7]. Although initial large-scale studies did not demonstrate significant benefits from incorporating AST-120 into the standard therapy for CKD patients, subsequent sporadic studies suggest potential support for its utilization [9, 10].

The clinical trials about AST-120 for uremic pruritus are few. Based on the evidences above, we supposed that the protein bounded uremic toxin might contribute to the uremic pruritus. The aim of the study was to assess if uremic pruritus could be mitigated by AST-120.

## MATERIALS AND METHODS

### Study design, protocol, and ethics

**Figure 1:**
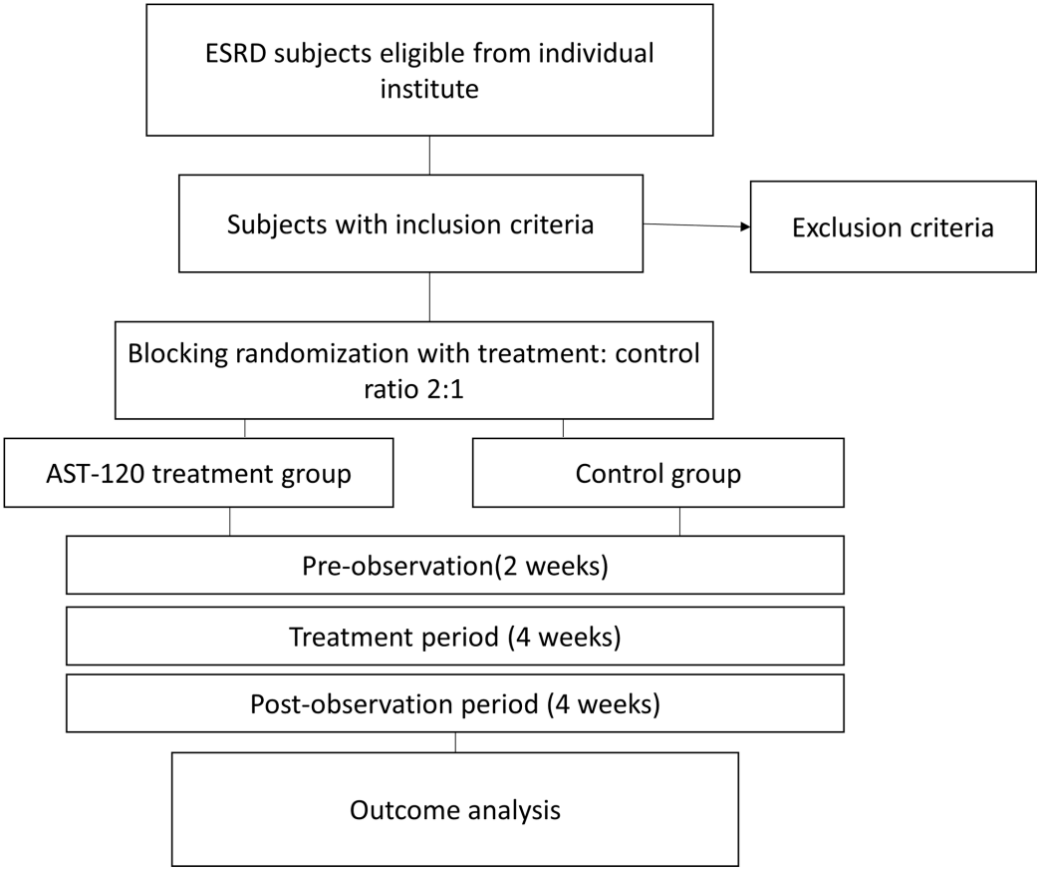
**The flow chart of the study.** Abbreviation: ESRD: end stage renal disease.

**Figure 2:**
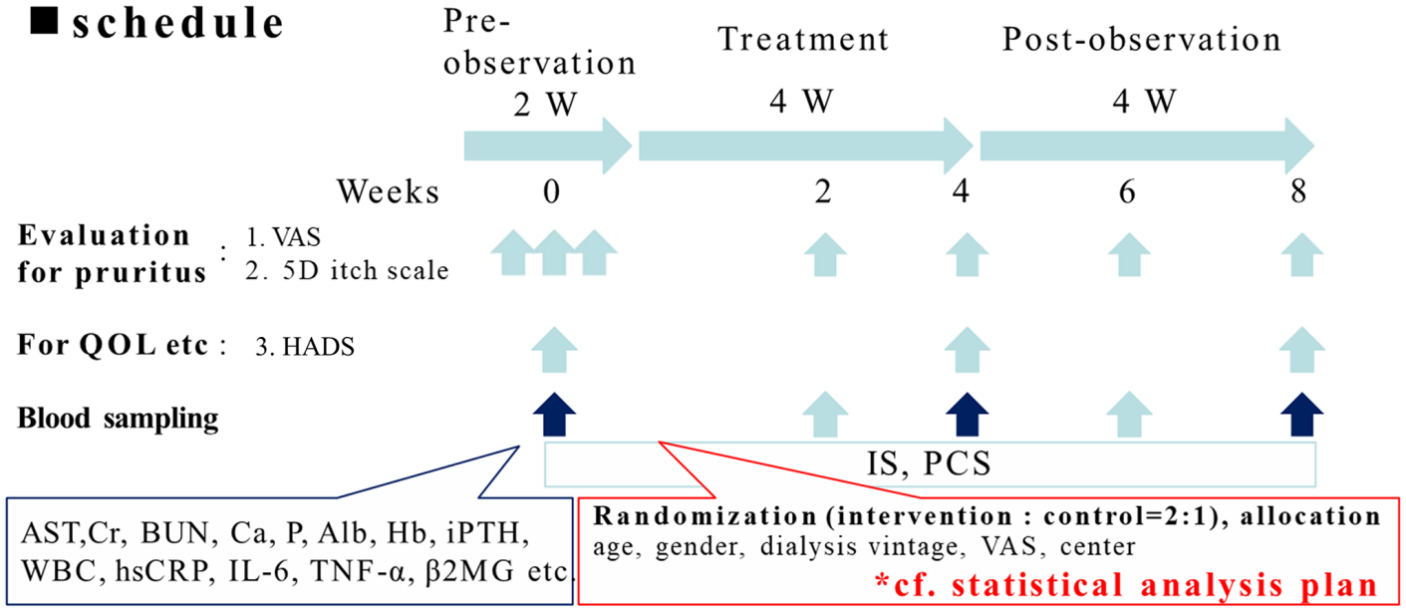
**The protocol of the clinical trial.** The study was divided into three phases: (1) a pre-observation phase of two weeks; (2) a treatment phase of four weeks; (3) a post-observation phase of four weeks. All the participants received pre-observation screenings two weeks before the study with three visits. In the treatment phase, the control group received oral or parental antihistamine or topical cream/ointment for pruritus as routine anti-pruritic treatment according to the principle of each institute. For the intervention group added 6 g/day of AST-120 along with routine anti-pruritic treatment. In the treatment phase, the participants received two visits, in the 2nd and 4th weeks. In the post-treatment phase, the participants received two visits, in the 6th and 8th week.

Figure 1 demonstrated the flow chart of the study. This was an interventional, multicenter, randomized open-label clinical trial initiated in Taiwan (https://clinicaltrials.gov/ registration identifier: NCT04639674). The study was performed from October 1, 2019, to September 30, 2020. The study protocol was approved by the Institute of Research Boards of the individual research sites following the tenets of the Declaration of Helsinki (Tri-Service hospital: 1-108-05-157; Taichung Veterans General Hospital: CG20082B; Tungs' Taichung Metroharbour Hospital: 108034; Chang Gung Memorial Hospital: 201901505A3; Kaohsiung Medical University Hospital: KMUHIRB-F(II)-20200010; Taipei Tzu Chi Hospital: 10-FS-039; Fu Jen Catholic University Hospital: FJUH110112). The study subjects were patients receiving maintenance hemodialysis continuously for more than three months. The study protocol is illustrated in Figure 2. The study was divided into three phases: (1) a pre-observation phase of two weeks; (2) a treatment phase of four weeks; and (3) a pro-observation phase of four weeks. The written inform consents were obtained from the participants after being explained by the trained stuff of the study group.

All participants underwent pre-observation screenings two weeks before the study with three visits. In the treatment phase (week 1 to week 4). In the treatment phase, the control group received oral or parental antihistamine or topical cream/ointment for pruritus as routine anti-pruritic treatment according to the principle of each institute. The intervention group added 6g/day of AST-120 along with routine anti-pruritic treatment. The participants received two visits at the 2nd and 4th weeks. In the post-observation phase (week 5 to week 8), the participants received two visits, at the 6th and 8th week. The participants underwent evaluation of pruritus during each visit. Assessment of the quality of life was performed in week 1 of the pre-observation screenings, week 4 of the treatment phase, and week 8 of the post-observation phase. Blood sampling was performed in the 1st week of the pre-observation phase and during all visits in the treatment phase and post-observation phases.

### Patients

This study targeted hemodialysis patients with severe uremic pruritis. The subjects would not enter the screening phase if they had the following conditions: (1) significant alteration in regimen within two weeks of the screening; (2) new or changes of treatment received for pruritus within two weeks of the screening; (3) having received ultraviolet treatment or acupuncture within six months of the screening; (4) having taken oral or intravenous antibiotics within one month of the screening period; (5) having participated or planning to participate in another clinical trial within two months of the screening; (6) alcohol or drug abuse within 12 months of the screening; and (7) a history of mental illness or poor drug compliance.

The treatment-to-control ratio in the screening phase was 2:1 with a randomized open-label clinical trial. The inclusion criteria were as follows: (1) ≥ 20 years of age, <100 years of age; (2) has been undergoing hemodialysis three times a week for at least six months (with Kt/V ≥1.2); (3) has continued anti-pruritic treatment (such as oral or intravenous antihistamine or topical cream/ointment for pruritus) for at least three months; (4) AST-120-naïve user; (5) mean visual analog scale (VAS) ≥4 three times in the last week of the pre-observation period; and (6) adequate venous access (has stable functioning arteriovenous fistula, graft, or other venous access).

The exclusion criteria were as follows: (1) precautions or special warnings for AST-120; (2) malignant hypertension, liver disease, cholestasis, heart disease (congestive heart failure, coronary artery disease, ischemic heart disease), stroke, malignant tumor, any acute illness, active infection, significant pulmonary disease; (3) dermatologic disease not due to uremic status, such as allergy or infection like tinea (diagnosed by a dermatologist, if necessary); (4) Ca >10.5 mg/dl or *P* > 6.5 mg/dl or Hb <9 g/dl or iPTH >600 pg/ml; (5) pregnancy or breastfeeding; (6) received oral or parental antibiotics before and throughout the study because antibiotics reduce the production of uremic toxins in an extreme manner; (7) not eligible to participate in this trial as per the researchers’ decision.

### Efficacy endpoints

#### Primary endpoint: evaluation of pruritus

The severities of uremic pruritus were measured by the visual analog scale (VAS) and 5-D itch scale. The VAS consisted of a 10-cm horizontal line with 0 points (no pruritus) to 10 points (maximum intensity of pruritus) and was given to patients after hemodialysis [11]. The VAS score was used as the tool of enrollment (as inclusion criteria 5). Uremic pruritus is defined as pruritus lasting for longer than three months with a VAS score of 4 or more (where 0 indicates no pruritus and 10 unbearable pruritus) [12]. The patients answer the VAS questionnaire by only considering the last month. The 5-D itch scale is a reliable, multidimensional measure of itching that has been validated in patients with chronic pruritus to be able to detect changes over time [13]. The assessment of pruritus was performed at each visit during the study.

#### Secondary endpoint: psychiatric morbidity assessment

This study also checked the Hospital Anxiety and Depression Scale (HADS) to assess the quality of life [14]. The HADS is a self-assessment scale for detecting anxiety and depression. The scale has a total score of 21, with anxiety and depression subcategories that have maximal scores of 12 and 9, respectively. A score from 0 to 7 is defined as normal; 8 to 10 as a borderline case with anxiety/depression; and 11 to 21 as a case with anxiety/depression. The quality-of-life assessment was performed at the 3rd, 5th, and 7th visits.

#### Secondary endpoint: laboratory measurements for protein-bound uremic toxins and routine parameters

All clinical parameters were measured at a central laboratory. The serum levels of total IS and PCS concentrations were measured in all patients by using LC-MS/MS. The following baseline demographic and clinical data were recorded: age, sex, height, weight, body mass index (BMI), causes of ESRD, comorbidities, systolic blood pressure (BP), diastolic BP, laboratory investigations, and therapeutic characteristics. Blood samples were drawn to measure serum hemoglobin, albumin, creatinine, blood urea nitrogen, potassium, total cholesterol, calcium, phosphorous, high-sensitivity C-reactive protein, intact parathyroid hormone (iPTH), and β2-microglobulin. For iPTH measurements, plasma samples were obtained and analyzed by electrochemiluminescence assay (Cobas e601; Roche Diagnostics GmbH, Mannheim, Germany). For iFGF23, plasma samples were analyzed by enzyme-linked immunosorbent assay (human FGF23 (intact); Immutopics Inc., San Clemente, CA, USA).

### Statistical analysis

The descriptive statistics are shown as numbers and percentages for counted numerical variables (such as gender), and means ± standard deviations for measured numerical variables (such as age, calcium, and albumin value). Spearman’s correlation was performed to correlate the variation between the biochemical parameters and the VAS score. Independent *t*-test was used to compare the difference in numerical variables between the AST-120 treatment group and control group. To compare the difference in the numerical variables of the pre- and post-treatment, paired *t*-test was used. Chi-square test was performed to compare the num The SPSS software program for Windows (ver. 20.00; SPSS Inc., Chicago, IL, USA) was used for data evaluation and statistical analysis.

### Data availability statement

The data that support the findings of this study are available from the corresponding author K-C Lu, upon reasonable request.

## RESULTS

### The demographic and baseline hematologic/biochemical parameters between the groups

Table 1 illustrates the demographic and baseline characteristics between the AST-120 treatment group (*n* = 68) and the control group (*n* = 31). The age, gender, and underlying illnesses such as hypertension or diabetes mellitus were similar between the groups. The hematologic and biochemical parameters, such as pre-dialytic blood urea nitrogen, creatinine, or other parameters, were similar between the groups.

**Table 1:** Demographic and baseline hematologic/biochemical results between the groups.

	**AST-120 treatment group**	**Control group**	***P*-value**
*Demographics*			
Age (years)	61.09 ± 12.15	65.31 ± 12.30	0.118
Sex (M/F)	49/19	19/11	0.531
Hypertension (-/+)	19/49	10/21	0.842
Diabetes mellitus (-/+)	24/44	15/16	0.310
*Biochemical parameters*			
BUN (mg/dL)	69.93 ± 20.56	70.84 ± 13.92	0.805
Creatinine(mg/dL)	10.66 ± 1.88	11.05 ± 2.84	0.511
Na (mEq/L)	136.23 ± 3.32	136.71 ± 3.58	0.591
K (mEq/L)	4.58 ± 0.69	4.62 ± 1.35	0.859
GOT (U/L)	14.74 ± 5.93	14.48 ± 5.90	0.867
Albumin (g/L)	4.01 ± 0.35	3.95 ± 0.29	0.410
Ca (mg/dL)	8.86 ± 1.07	8.89 ± 0.90	0.918
P (mg/dL)	4.78 ± 1.27	4.69 ± 1.29	0.752
Hemoglobin (g/dL)	10.52 ± 1.01	10.94 ± 1.14	0.070
White cell blood count (1000/μl)	6.75 ± 2.03	7.02 ± 2.05	0.614

### The severity of pruritus decreased in the AST-120 treatment group

Table 2 illustrates the severity of the uremic pruritis between the AST-120 treatment group and the control group. 3 participants of AST-120 treatment group dropped out due to refusal for continuing the study. The mean values of the VAS score, 5-D itch scale score, and HADS depression anxiety score (A score) and score (D score) were similar between the groups in the first visit (for the AST-120 group: (1) VAS: 7.14 ± 1.75; (2) 5-D itch scale score: 17.80 ± 4.43; (3) D score: 4.58 ± 3.32; (4) A score: 4.22 ± 3.74. For the control group: (1) VAS: 6.75 ± 1.58; (2) 5-D itch scale score: 18.32 ± 5.67; (3) D score: 4.97 ± 3.86; (4) A score: 4.10 ± 4.09). The VAS score began to be lower in the AST-120 treatment group after the 5th visiting (5th: 4.35 ± 2.13 vs. 6.18 ± 1.69; 6th: 4.21 ± 2.11 vs. 5.86 ± 1.63; 7th: 4.15 ± 2.25 vs. 5.57 ± 1.93; *p* < 0.05, respectively). The 5-D itch scale score began to be lower in the AST-120 treatment group after the 4th visit (4th: 14.75 ± 4.06 vs. 17.74 ± 5.83; 5th: 12.48 ± 3.40 vs. 16.77 ± 6.73; 6th: 12.66 ± 5.10 vs. 16.90 ± 6.30; 7th: 16.90 ± 6.30 vs. 16.53 ± 6.92; *p* < 0.05, respectively). The D score and A score were similar between the groups in each visit. Figure 2 illustrated the VAS score and 5-D itch scale score between AST-120 and control group.

**Table 2:** Visual analog scale (VAS), 5-D itch scale, and hospital anxiety and depression scale (HADS) depression score (D score) and anxiety score (A score) at each visit according to the protocol.

	**AST-120 treatment group (*n* = 65)**	**Comparison with baseline**	**Control group (*n* = 31)**	**Comparison with baseline**	**Comparison between AST-120 treatment and control groups**
	**Mean (SD)**	***P*-value**	**Mean (SD)**	***P*-value**	***P*-value**
VAS 1st visit	7.08 (1.73)		6.72 (1.56)		0.331
2nd	6.76 (1.65)	1.000	6.56 (1.47)	1.000	0.362
3rd	6.70 (1.70)	0.709	6.61 (1.51)	1.000	0.460
4th	5.52 (2.01)	<0.001^*^	6.49 (1.49)	1.000	0.069
5th	4.43 (2.09)	<0.001^*^	6.21 (1.72)	1.000	<0.001^*^
6th	4.21 (2.11)	<0.001^*^	5.88 (1.65)	0.039^*^	0.001^*^
7th	4.15 (2.25)	<0.001^*^	5.73 (1.75)	0.045^*^	0.002^*^
5-D 1st visit	17.67 (4.53)		18.59 (5.77)		0.708
2nd	17.40 (4.42)	1.000	17.76 (5.20)	0.456	0.726
3rd	17.09 (4.54)	1.000	17.86 (5.03)	1.000	0.802
4th	14.32 (3.54)	<0.001^*^	17.83 (5.99)	1.000	0.006^*^
5th	12.60 (3.42)	<0.001^*^	17.00 (6.73)	0.343	0.002^*^
6th	12.65 (5.14)	<0.001^*^	16.97 (6.40)	0.665	<0.001^*^
7th	12.51 (5.10)	<0.001^*^	16.76 (6.93)	0.786	0.002^*^
D score 3rd visit	4.47 (3.30)		5.07 (3.99)		0.759
5th	2.82 (2.63)	<0.001^*^	4.29 (3.67)	0.044^*^	0.061
7th	3.05 (3.18)	0.001^*^	3.71 (3.67)	0.017^*^	0.518
A score 3rd visit	4.57 (3.84)		4.29 (4.25)		0.660
5th	4.16 (3.58)	0.730	3.96 (3.89)	1.000	0.652
7th	3.34 (3.16)	0.010^*^	3.79 (3.92)	0.847	0.824

Abbreviation: SD: Standard Deviation). ^*^p < 0.05.

### The concentration of PBUT and inflammatory cytokines decreased in the AST-120 treatment group

**Figure 3:**
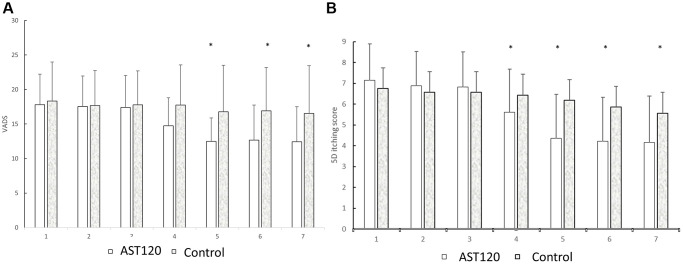
The comparison of the VADS (**A**) and 5D itch scores (**B**) between the AST-120 group and control group from the 1st to 7th visit. ^*^p < 0.05, AST-120 vs. control group at each visit.

Table 3 shows the concentration of uremic toxins, HS-CRP, interleukin-6, TNF-alpha, and β2-microglobulin at the 3rd, the 5th, and 7th visits between the groups. The concentration of IS, PCS, HS-CRP, interleukin-6, TNF-alpha, and β2-microglobulin were similar at the 3rd visit between the groups (IS: 42.81 ± 2.42 μg/mL vs. 49.22 ± 5.17 μg/mL; PCS: 32.90 ± 3.16 μg/mL vs. 35.35 ± 4.01 μg/mL; HS-CRP: 5.40 ± 0.89 mg/L vs. 5.00 ± 1.31 mg/L; IL-6: 9.23 ± 2.14 pg/mL vs. 6.15 ± 0.65 pg/mL, iPTH (pg/mL): 272.02 ± 29.43 vs. 209.67 ± 40.83; β2-microglobulin: 19033.93 ± 726.65 ng/mL vs. 21179.50 ± 1186.66 ng/mL; TNF-alpha: 2.67 ± 0.20 pg/mL vs. 2.78 ± 0.24 (pg/mL)). In the AST-120 treatment group, the concentration of IS, PCS, and TNF-alpha became lower after the 4th visit (IS: 4th visit: 32.87 ± 2.00 pg/mL; 5th visit: 35.68 ± 2.09 pg/mL; *p* < 0.05; PCS: 4th visit: 26.65 ± 2.74 pg/mL; 5th visit: 29.22 ± 2.90 pg/mL; *p* < 0.05). The TNF-alpha was lower in the 5th (2.32 ± 0.12 pg/mL) and 7th visit (2.26 ± 0.10 pg/mL). Figure 3 illustrated the concentration change in indoxyl sulfate, p-cresol sulfate and TNF-alpha when comparing with 3rd visit in AST-120 and control group.

**Table 3:** Comparison between protein-bound uremic toxins and other parameters.

	**AST-120 group Mean (SD)**	**Comparison with baseline (*p*-value)**	**Control group Mean (SD)**	**Comparison with baseline (*p*-value)**	**Comparison between AST-120 and control groups**
HS-CRP (mg/L) 3rd visit	5.40 (0.89)		5.00 (1.31)		0.359
5th	5.82 (1.12)	0.726	6.04 (1.96)	0.621	0.684
7th	6.15 (1.21)	0.379	4.59 (1.36)	0.784	0.229
IL-6 (pg/mL) 3rd visit	9.23 (2.14)		6.15 (0.65)		0.493
5th	6.35 (0.56)	0.185	7.26 (1.07)	0.256	0.382
7th	13.93 (6.00)	0.459	7.68 (1.20)	0.148	0.435
Indoxyl sulfate (μg/mL) 3rd visit	42.81 (2.42)		49.22 (5.17)		0.473
4th	32.87 (2.00)	<0.001^*^	48.96 (5.26)	0.874	0.005
5th	35.68 (2.09)	<0.001^*^	49.95 (5.10)	0.754	0.028
6th	39.93 (2.44)	0.074	54.36 (5.11)	0.059	0.021
7th	38.92 (2.77)	0.066	48.56 (4.86)	0.799	0.143
iPTH (pg/mL) 3rd visit	272.02 (29.43)		209.67 (40.83)		0.091
5th	287.49 (35.50)	0.320	212.57 (46.17)	0.834	0.066
7th	293.30 (35.54)	0.257	235.82 (47.69)	0.149	0.182
p-cresyl sulfate (μg/mL) 3rd visit	32.90 (3.16)		35.35 (4.01)		0.805
4th	26.65 (2.74)	<0.001^*^	33.86 (4.05)	0.390	0.305
5th	29.22 (2.90)	0.036^*^	34.76 (4.65)	0.773	0.472
6th	32.18 (2.99)	0.662	37.86 (4.00)	0.325	0.295
7th	30.76 (3.07)	0.234	35.76 (4.05)	0.899	0.263
β2-Microglobulin (ng/mL) 3rd visit	19033.93 (726.65)		21179.50 (1186.66)		0.277
5th	18442.88 (669.23)	0.379	20683.35 (1044.61)	0.671	0.065
7th	19014.90 (638.65)	0.974	21460.19 (1169.35)	0.784	0.058
TNF-alpha (pg/mL) 3rd visit	2.67 (0.20)		2.78 (0.24)		0.624
5th	2.32 (0.12)	0.007^*^	2.54 (0.18)	0.140	0.283
7th	2.26 (0.10)	0.014^*^	2.43 (0.18)	0.044	0.301

Abbreviations: HsCRP: High sensitive C-reactive protein; IL-6: interleukin 6; iPTH: intact parathyroid hormone; SD: standard deviation; TNF-alpha: tumor necrosis factor alpha.

### The correlation between the reduction of indoxyl sulfate and the variation of inflammatory cytokine, parathyroid hormone, and the VAS score

Table 4 shows a comparison of reduction ratio (5th vs. 3rd week and 7th vs. 3rd week) of the individual parameters between the AST-120 treatment group and the control group. The reduction ratio was determined as: (100% × (the concentration of the week) − (the concentration of 3rd week)/the concentration of 3rd week). The reduction ratio of indoxyl sulfate at 5th week was lower than the control group. At 7th week, the reduction ratio of indoxyl sulfate was similar between group. The reduction of ratio of other parameters were similar between the AST-120 treatment group and control group.

**Table 4:** Reduction ratio of pre-and post-treatment values between the AST-120 treatment group and the control group (5th vs. 3rd week and 7th vs. 3rd week).

	**5th vs. 3rd week (AST-120 treatment group)**	**5th vs. 3rd week (control group)**	***P*-value**	**7th vs. 3rd week (AST-120 treatment group)**	**7th vs. 3rd week (control group)**	***P*-value**
HsCRP (%)	83.14 ± 43.13	133.08 ± 91.60	0.575	69.29 ± 29.78	56.39 ± 28.04	0.790
IL-6 (%)	12.07 ± 10.47	32.39 ± 14.46	0.269	126.67 ± 112.28	35.42 ± 14.89	0.589
Indoxyl sulfate (%)	−13.98 ± 4.38	3.85 ± 6.11	0.021^*^	−5.89 ± 5.44	7.23 ± 9.28	0.200
iPTH (%)	26.46 ± 16.18	10.62 ± 9.39	0.524	36.18 ± 15.17	38.89 ± 19.78	0.918
p-cresyl sulfate (%)	5.35 ± 10.98	6.43 ± 12.83	0.953	21.77 ± 19.41	22.14 ± 17.83	0.990
β2-microglobulin (%)	4.56 ± 4.70	5.88 ± 8.24	0.882	6.21 ± 4.33	6.68 ± 6.96	0.953
TNF-alpha (%)	−8.38±2.74	−5.14±3.84	0.502	−8.04±3.35	−7.31±4.78	0.902

^*^p < 0.05. Data presented as Mean ± Standard Deviation (SD). Abbreviations: HsCRP: High sensitive C-reactive protein; IL-6: interleukin 6; iPTH: intact parathyroid hormone; SD: standard deviation; TNF-alpha: tumor necrosis factor alpha.

Table 5 demonstrates the correlation between IS reduction and the reduction of other parameters. The reduction of PCS was associated with the reduction of IS at the 5th and 7th visits (*p* < 0.05). The reduction of iPTH, on the other hand, correlated with the reduction of IS at the 7th visit (*p* < 0.05).

**Table 5:** Spearman correlation between the reduction ratio between indoxyl sulfate and other parameters in the AST-120 treatment group.

	**5th week**	***P*-value**	**7th week**	***P*-value**
HsCRP	−0.06	0.668	−0.24	0.06
IL-6	−0.24	0.061	−0.19	0.146
iPTH	0.11	0.394	0.31	0.016^*^
p-cresyl sulfate	0.52	<0.001^*^	0.29	0.024^*^
β2-microglobulin	0.08	0.522	0.12	0.378
TNF-alpha	−0.06	0.634	−0.07	0.575

^*^p < 0.05. Abbreviations: HsCRP: High sensitive C-reactive protein; IL-6: interleukin 6; iPTH: intact parathyroid hormone; TNF-alpha: tumor necrosis factor alpha.

Table 6 shows the reduction of the VAS and other parameters in the AST-120 treatment group. The lowering of IS was marginally correlated with the lowering of the VAS lowering at the 5th visit (*p* = 0.053).

**Table 6:** Correlation between the variation of the VAS and variations of other parameters in the AST-120 treatment group.

	**Spearman correlation coefficient**	***P*-value**
5th visit - 3rd visit		
HsCRP	−0.04	0.783
IL-6	−0.14	0.305
Indoxyl sulfate	0.25	0.053
iPTH	−0.17	0.194
p-cresyl sulfate	0.13	0.330
β2-Microglobulin	−0.16	0.231
TNF-alpha	−0.13	0.342
7th - 3rd visit		
HsCRP	0.04	0.760
IL-6	−0.13	0.347
Indoxyl sulfate	-0.14	0.298
iPTH	−0.01	0.969
p-cresol sulfate	0.10	0.452
β2-Microglobulin	0.04	0.767
TNF-alpha	0.10	0.472

### The impact of AST-120 treatment on VADS and 5D itch scores

Figure 3 presents a comparison of VADS and 5D itch scores between the AST-120 group and the control group from the 1st to the 7th visit. (A) Following the 5th visit, the AST-120 treatment resulted in a significant decrease in VADS scores. (B) However, in the case of 5D itching scores, the AST-120 treatment exhibited a significant attenuation starting from the 4th visit.

### The effect of AST-120 treatment on uremic toxins and inflammatory cytokine levels

**Figure 4:**
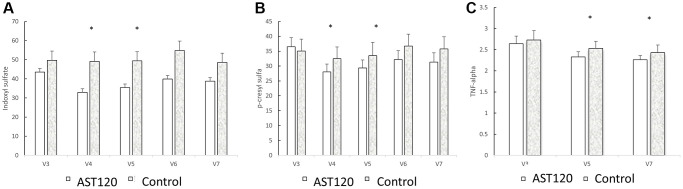
The comparison of indoxyl sulfate (**A**), p-cresol sulfate (**B**), and TNF-α (**C**) levels between the AST-120 group and the control group from the 3rd to the 7th visit. ^*^p < 0.05, when comparing 3rd visit for AST-120 group.

Figure 4 illustrates the comparison of indoxyl sulfate, p-cresol sulfate, and TNF-α levels between the AST-120 group and the control group from the 3rd to the 7th visit. AST-120 treatment led to a significant decrease in indoxyl sulfate (A) and p-cresol sulfate (B) levels at the 4th and 5th visits. Furthermore, TNF-α (C) levels exhibited a significant decrease in the 5th and 7th visits.

## DISCUSSION

This study has demonstrated that the four-week treatment of AST-120 decreased the severity of uremic pruritis in the ESRD patients in the VAS and 5-D itch scale. AST-120 lowered the serum concentration of IS, PCS, and TNF-alpha after four weeks of treatment. When comparing the absolute values with the subjects without AST-120 treatment, IS (at the 3rd, 4th, and 5th visits) and β2-microglobulin (at the 5th visit) were lower in the AST-120 treatment group.

### The treatment of AST-120 decreased the severity of pruritus in the ESRD patients

The pathogenesis of uremic pruritis is still poorly understood, but the histamine-dependent and independent neurons are essential for transmitting the itch signal to the central nervous system. Traditionally, the activation of histamine-dependent mechanically-insensitive C-fibers is the major pathogenesis for the development of pruritis. The histamine receptor sensitizes TRPV1 and transmits the itch sensation through the peripheral neurons [15]. Antihistamine therapeutics targeting histamine receptor 1 provide relief of pruritus [16]. However, non-histamine-dependent polymodal C-fibers would be activated in the epidermis with further activating protease-activated receptor 2 in the peripheral terminal [17]. The stimuli by pruritogen or noxious agents transmit the signal by spinothalamic tract neurons and further format the pruriceptive sensation [18], and therefore antihistamine agents provide insufficient efficacy for subjects with impaired somatosensory sensation [19].

Beyond mitigating the activation of histamine or non-histamine dependent signals, removal of pruritogen should be an important strategy based on the pathogenesis of pruritus. Clinical studies have demonstrated that the concentration of total PCS was associated with the development of pruritis in CKD patients, even after adjusting for other well-known contributing factors [6]. In CKD patients or ESRD patients with residual function, the residual function of the organic anion transporter could facilitate the excretion of the IS or other protein-bound uremic toxins (PBUTs) [20]. The clearance of PBUTs was insufficient in the conventional dialysis modality [21]. Polymethylmethacrylate (PMMA)-based dialysis membranes, which remove IS or PCS with moderate efficacy, also provided moderate efficacy in alleviating the pruritic sensation, but the role in lowering PBUTs and inflammatory cytokines was uncertain [22]. The strategy for lowering protein-bound uremic toxins has been suggested for uremic pruritus. Tseng et al. demonstrated that a vegetarian diet ameliorated uremic pruritis in dialysis patients along with lowering the severity of inflammation [23]. The strategies for lowering the burden of uremic toxins by intestinal removal should be a potential therapeutic target in mitigating uremic pruritus. Niwa et al. demonstrated that AST-120 decreased the body burden of IS and pruritis in dialysis patients [8]. AST-120 is an oral spherical carbonaceous adsorbent for intestinal clearance of the precursor of uremic toxins, especially protein-bound uremic toxin [24]. Previous studies have demonstrated the efficacy of AST-120 in lowering the burden of uremic toxins and glomerular filtration rate decline or associated uremic symptoms in non-dialysis patients [9, 25]. However, the application for dialysis patients is scant. To our understanding, this study is the first study demonstrating the therapeutic role of AST-120 in dialysis patients. The therapeutic duration in the study was four weeks, which was similar to other clinical trials for lowering uremic toxins by other modalities [22].

### The treatment of AST-120 decreased the severity of pruritus in ESRD patients via lowering inflammatory cytokines

The mechanisms for uremic pruritis include skin alteration, inflammation, nociceptive receptor dysfunction, and opioid receptor dysfunction. Recently, Moon et al. noticed the activation of protease-activated receptor 2 within the epidermal keratinocyte [26]. From our results, the level of TNF-alpha decreased after four weeks of AST-120 treatment along with an improvement of the severity of uremic pruritus. Systemic inflammation has been proposed as possible pathogenesis for uremic pruritus. Previous studies have demonstrated that the serum concentration of pro-inflammatory cytokines, such as interleukin-6 or 2, is higher in ESRD patients with pruritus [27, 28]. Inducible nitric oxide synthase (iNOS) plays an important role in inflammation-mediated pruritus. Miyachi et al. have demonstrated that TNF-alpha activates the iNOS expression in the keratinocyte, and ultraviolet B radiation, which has been regarded as a treatment for refractory uremic pruritus, lowers the iNOS expression [29]. Toll-like receptors are the recognized receptors for innate immunity, and the activation of toll-like receptors could promote the production of iNOS by myeloid differentiation [30, 31]. Toll-like receptors 2 and 4 on the macrophage govern the activation of iNOS released during the inflammatory process, and IS and PCS are potential promoters for inducing the toll-like receptors [32, 33]. We have noticed that the concentration of IS and PCS decreased after two weeks of AST-120. The concentration of protein-bound uremic toxins would increase to the pre-treatment level in the 3rd and 4th weeks. A decrease of TNF-alpha occurred in the 5th and 6th weeks of treatment. Our data also first demonstrated the time course of protein-bound uremic toxins and cytokines in hemodialysis patients. The analysis of antigen-presenting cells such as macrophages could be a future perspective for understanding the effect of uremic toxins and uremic pruritis.

### The reduction of indoxyl sulfate might be associated with the lowering of PTH in ESRD patients along with the lowering of PTH with AST-120 treatment

Our study has demonstrated that the lowering of IS posed a marginal effect on the improvement of the VAS along with the lowering of serum concentration of iPTH. PTH plays a role in modulating the differentiation of keratinocytes. *In vitro*, studies have demonstrated PTH could diverge the proliferating process of the keratinocyte into terminal differentiation [34, 35]. Epidermal skin atrophy is observed in uremic pruritus, and therefore lowering the PTH might facilitate the regenerative ability of keratinocytes [36]. Several kinds of literature have discussed the interaction between IS and PTH. The simultaneous lowering of PTH with IS has been demonstrated in dialysis patients receiving a low-phosphorus-to-protein ratio [37]. PTH is essential for bone remodeling by activating the signal transduction on the osteoblasts. IS could impair the bone remodeling via the sensitivity of PTH on the osteoblasts and the RANKL-dependent differentiation of osteoclasts [38, 39]. Therefore, the reduction of IS might provide a conjunctive role in the management of mild to moderate secondary hyperparathyroidism (iPTH ≤600 pg/mL) in hemodialysis patients. Our study has demonstrated a possible causality of the IS and PTH in uremic pruritus.

This study still has several limitations. First, the treatment period was limited to four weeks in the AST-120 treatment group. The duration of AST-120 treatment is generally from 12 to 108 weeks [25, 40, 41]. The sustained efficacy of AST-120 could not be demonstrated by the current study design. Second, a larger sample size could evaluate the efficacy of AST-120 on ESRD patients with different entities. Third, a comparison between the severity of pruritis among responders and non-responders in the AST-120 treatment group was not performed in this study. Comparing the severity between responders and non-responders might provide further understanding of uremic pruritus.

In summary, this study has demonstrated that the four-week treatment of AST-120 decreased the severity of pruritus in maintenance hemodialysis patients. Along with the severity of uremic pruritus, the concentration of IS and PCS decreased in the AST-120 treatment group. A decrease in TNF-alpha and β2-microglobulin also developed in the AST-120 treatment group. The IS lowering was associated with lowering of parathyroid hormone.
